# Targeted therapy in head/neck and gastric cancers

**DOI:** 10.1186/1756-8722-5-S1-A1

**Published:** 2012-04-25

**Authors:** Chung-Tsen Hsueh

**Affiliations:** 1 Loma Linda University, Loma Linda, CA 92354, U.S.A

## 

The epidermal growth factor receptor (EGFR/HER1) is a member of the erbB family of receptor tyrosine kinase proteins, which also includes HER2, HER3, and HER4. EGFR is almost universally expressed in squamous cell carcinoma of head and neck (SCCHN), and high levels of expression have been correlated with a poor clinical prognosis [1]. Cetuximab, an IgG1 monoclonal antibody against EGFR, has demonstrated improved survival and disease control when used in combination with radiation therapy for the treatment of locally advanced SCCHN, and in combination with platinum-based chemotherapy in recurrent or metastatic SCCHN [2-4]. Additionally, single-agent cetuximab is active and provides good disease control rate and duration in platinum-refractory SCCHN [5]. Comparison of cetuximab versus cisplatin concurrently with radiotherapy is under investigation in patients with human papillomavirus-associated oropharyngeal cancer, who have better prognosis and may benefit from less toxic treatment [6].

Overexpression of HER2 in gastric cancer results in aggressive clinical course and poor prognosis [7]. Trastuzumab, a monoclonal antibody against HER2, exhibits antitumor activity in HER2 overexpressed gastric cancer cells, and enhances effects of chemotherapy in gastric cancer xenograft overexpressing HER2 [8]. The ToGA study screened about 3,800 patients with advanced gastric cancer from 24 countries, and HER2 overexpression was detected in 22% [9]. Higher rates of HER2 overexpression occurred in intestinal and proximal or gastroesophageal junction cancers than in diffuse or distal gastric cancers. In TOGA study, 584 patients with HER2 overexpression were randomized to receive fluoropyrimidine and cisplatin treatment with or without trastuzumab. Patients who received trastuzumab plus chemotherapy achieved longer overall survival (13.8 months vs. 11.1 months, P= 0.0046), longer progression-free survival (6.7 months vs. 5.5 months, P=0.0002), and higher response rates (47% vs. 35%, P=0.0017) than those who received chemotherapy alone. Complete response was noted in 5.4% of patients receiving trastuzumab plus chemotherapy vs. 2.4% in chemotherapy alone. There were no significant differences in the toxicities between these two groups. This study has established a new paradigm using trastuzumab in combination with chemotherapy in patients with advanced gastric cancer overexpressing HER2.

Neoadjuvant treatment is a standard of care for locally advanced esophageal and gastric cancer. We have previously reported a case of pathological complete response after neoadjuvant chemotherapy with trastuzumab-containing regimen in HER2-overexpressing gastric cancer [10]. Incorporating trastuzumab as a part of neoadjuvant therapy in esophageal and gastric adenocarcinoma overexpressing HER2 is currently under active investigation. Radiation Therapy Oncology Group is conducting a phase III neoadjuvant study in patients with HER2-overexpressing esophageal adenocarcinoma to determine if trastuzumab increases disease-free survival when added to chemoradiotherapy [11]. Other studies conducted in Europe are adding trastuzumab to oxaliplatin-based regimen as perioperative chemotherapy for HER2-overexpressing esophagogastric or gastric adenocarcinoma, and looking for improvement of pathological complete response and disease-free survival [12,13].

Pertuzumab is a monoclonal antibody interfering with HER2 dimerization with other HER receptors such as EGFR, HER3 and HER4. Pertuzumab and trastuzumab bind to HER2 at different sites, and combination of both antibodies leads to stronger inhibition of erbB signaling and greater therapeutic efficacy when combined with docetaxel in breast cancer [14]. Combination of pertuzumab and trastuzumab with platinum-based chemotherapy is currently studied in HER2-overexpressing gastric cancer [15].
